# A natural language processing approach reveals first-person pronoun usage and non-fluency as markers of therapeutic alliance in psychotherapy

**DOI:** 10.1016/j.isci.2023.106860

**Published:** 2023-05-12

**Authors:** Jihan Ryu, Stephen Heisig, Caroline McLaughlin, Michael Katz, Helen S. Mayberg, Xiaosi Gu

**Affiliations:** 1 Department of Psychiatry, Icahn School of Medicine at Mount Sinai, New York, NY, USA; 2 Department of Neurology, Icahn School of Medicine at Mount Sinai, New York, NY, USA; 3 Department of Neuroscience, Icahn School of Medicine at Mount Sinai, New York, NY, USA; 4 Clinical Psychology Doctoral Program, School of Health Professions and Nursing, Long Island University - CW Post Campus, Greenvale, NY, USA

**Keywords:** Machine learning, Psychiatry

## Abstract

It remains elusive what language markers derived from psychotherapy sessions are indicative of therapeutic alliance, limiting our capacity to assess and provide feedback on the trusting quality of the patient-clinician relationship. To address this critical knowledge gap, we leveraged feature extraction methods from natural language processing (NLP), a subfield of artificial intelligence, to quantify pronoun and non-fluency language markers that are relevant for communicative and emotional aspects of therapeutic relationships. From twenty-eight transcripts of non-manualized psychotherapy sessions recorded in outpatient clinics, we identified therapists’ first-person pronoun usage frequency and patients’ speech transition marking relaxed interaction style as potential metrics of alliance. Behavioral data from patients who played an economic game that measures social exchange (i.e. trust game) suggested that therapists’ first-person pronoun usage may influence alliance ratings through their diminished trusting behavior toward therapists. Together, this work supports that communicative language features in patient-therapist dialogues could be markers of alliance.

## Highlights

• Usage of “i” and “we” in therapist speech characterizes sessions with low alliance
• Non-fluency in patient speech characterizes sessions with high alliance
• Diminished trusting behavior mediates the potential impact of therapists’ “i” usage

## Graphical abstract

**Figure:**
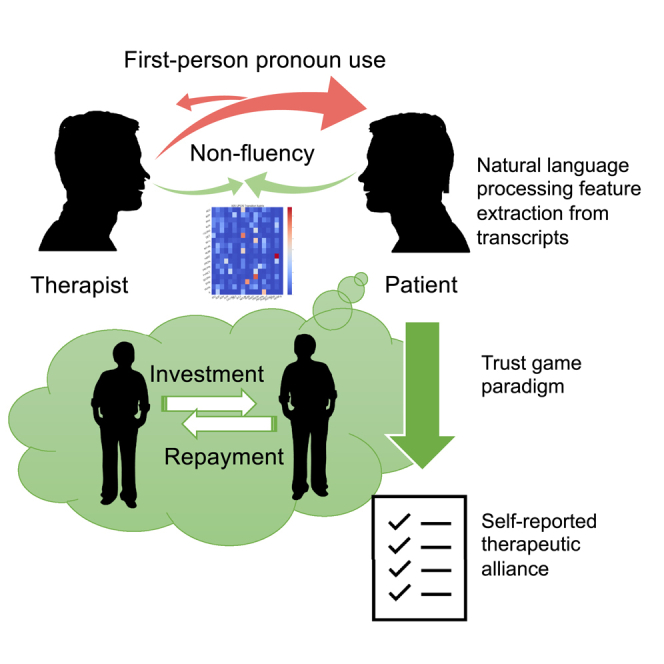

## Introduction

Therapeutic alliance, the collaborative and trusting quality of patient-clinician relationship, is an active ingredient of successful psychotherapy treatment outcomes including patient engagement, retention, and eventual symptom improvement.^1^ However, alliance has been notoriously hard to assess in real-world clinical practice due to conceptual and methodological challenges in finding objective markers that underlie patients’ subjective experience of closeness with clinicians. Current gold standard assessments of alliance rely on either self-reports or human observers’ qualitative coding of treatment interactions, which are subjective, labor-intensive, and time-consuming.^2^ These drawbacks have limited the development of a scalable, real-time feedback system to treatment pairs that can improve clinical outcomes through the timely identification of negative session-level alliance.^3^

One promising approach to leverage is the recent adoption of machine learning in health care research. For example, efforts have been made to assess alliance from a variety of measures directly recorded during therapy sessions such as patient-clinician language use,^4^^,^^5^ head and body movements,^6^ facial expressions,^7^ respiration rate and heart rate variability,^8^ and brain activities.^9^^,^^10^ These studies have found initial evidence that the behavioral and physiological synchrony between patients and therapists during sessions can be measured as a proxy of alliance.^4^^,^^6^^,^^7^^,^^8^^,^^9^ To be clinically actionable, however, data-driven approaches must identify what specific behavioral features clinicians can pay attention to and potentially adjust to optimize alliance during therapy. Much of existing computational work unfortunately fails to provide such interpretability. For example, several studies using natural language processing (NLP), a subset of artificial intelligence that learns data structure from human language, have revealed algorithms that could predict patient-rated alliance,^11^ therapist skills,^12^^,^^13^ and therapeutic rupture events from session transcripts.^14^ However, these algorithms are generally trained from the entire set of sentences uttered by a speaker and often provide high dimensional predictive features that are hard to interpret. Indeed, the lack of interpretability of model features in machine learning has been raised as a culprit for clinicians’ reluctance to utilize artificial intelligence in health care.^15^

In this study, we aimed to address this gap by combining both hypothesis- and data-driven approaches to assess personal pronoun usage and non-fluency in patients and therapists as interpretable linguistic markers of alliance. The rationale for pre-defining speech features this way is mainly 2-fold. First, the capacity of patients and therapists to communicate each other’s thoughts and emotions adaptively has been identified as a universal factor that impacts alliance and clinical outcomes across heterogeneous psychotherapy practice settings.^16^ Empirically, increased reference to the self in a dialogue—commonly represented by first-person singular personal pronouns, such as “I” and “me” - has been considered markers of failure to adaptively distance from negative emotional cues^17^ and internalizing symptoms in the text messages of patients in online therapy.^18^ The high frequency of self-focus through “I” usage has been generally linked with mental health burden, such as depression,^19^ post-traumatic stress disorder (PTSD),^20^ and compulsivity and intrusive thoughts.^21^ By contrast, the use of “I” as an active voice^22^ and an interactive agent with a therapist’s discourse^23^ were associated with positive therapy outcomes, suggesting the importance of engagement in a therapeutic dialogue. Second, relaxed styles during interactions have been observed as markers of highly affiliative relationships.^24^ For example, the frequency of filler pauses (“um”), indicating relaxed production of natural speech, was associated with multiple indices of high alliance interactions,^25^ such as a speaker’s truthfulness,^26^ emotional suppression,^27^ and increased attention during a storytelling task in healthy volunteers.^28^ Linguistic coordination in usage of the similar words and the rates at which they are said between two people have been shown to predict empathy, social support, and positive outcomes in individual therapy^29^^,^^30^^,^^31^ and online mental health support communities.^32^

Leveraging the feature extraction methods commonly used in NLP, here we quantified first-person pronouns and non-fluency as communicative function markers of both patients and therapists from single-session transcripts and regressed these features on post-session alliance scores rated by the subjects. As an additional proxy of alliance with the therapy partner, we also administered the trust game, a behavioral economics paradigm that quantifies trust and reciprocity between two people as they play the roles of an “investor” and a “trustee” during monetary exchange.^33^^,^^34^ Previous work has demonstrated that clinicians’ communication ability was positively associated with patients’ trust toward clinicians measured by how much patients would “repay” an investment in the trust game.^35^ We calculated the subjects’ repayment behavior toward therapy partner as an independent outcome variable of the session and tested if it would mediate the association between significant linguistic features and self-reported alliance. We hypothesized that the subject’s higher use of first-person pronoun usage would correlate with lower therapeutic alliance. In contrast, we hypothesized that the subject’s higher frequency of non-fluency markers, such as filler word usage would correlate with higher therapeutic alliance. Though findings in these speech features have been previously limited to patients or healthy controls in the laboratory, we hypothesized that the same direction of correlations will be observed in both patients’ and therapists’ language features, which together construct a treatment session.

## Results

### Alliance rating

Working alliance inventory—short form rated by patients ranged from 41 to 84 with mean score of 70 (SD 12). Patient-rated alliance was positively correlated with therapist-rated alliance scores (r = 0.56, p < 0.002) and alliance with the previous therapist (n = 27; 1 missing, r = 0.46, p = 0.02). Therapist-rated alliance was positively correlated with patient’s avoidant attachment scores (r = 0.40, p = 0.04). There were no differences in patient’s alliance across individual age, sex, diagnosis, attachment scores, duration of treatment, therapist’s experience, medium or modality of treatment (p > 0.05) (see Figure S1).

### Relationship between pronouns and alliance rating

**Figure 1:**
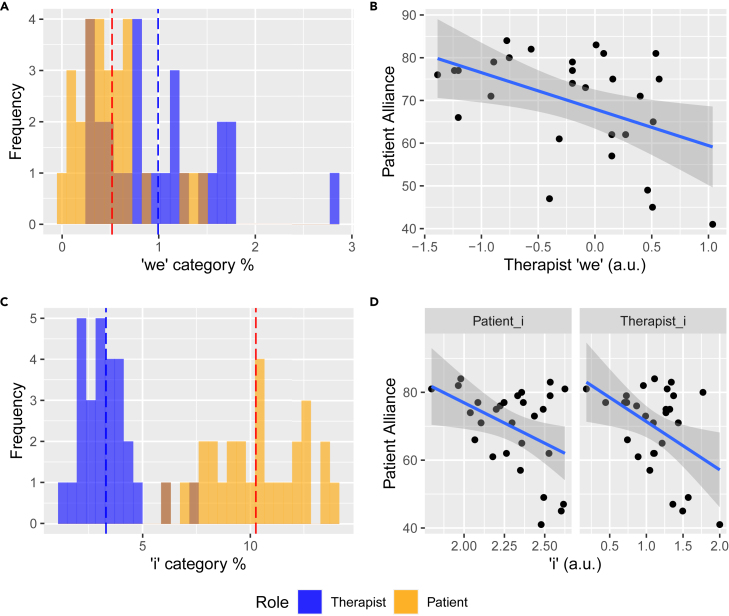
First-person pronoun frequencies negatively correlate with therapeutic alliance (A and C) Frequency distributions of “we” and “i” category features extracted from LIWC between speaker roles. Instances of each feature were divided by the total word count in their individual speech in the transcript. Dotted vertical lines indicate the group means (mean = 0.994, s.e. = 0.022 for “therapist_we”; mean = 0.517, s.e. = 0.013 for “patient_we”; mean = 3.30, s.e. = 0.047 for “therapist_i”; mean = 10.3, s.e. = 0.077 for “patient_i”). (B and D) First-person pronoun features show negative correlation with patient-rated alliance (r = −0.451, p = 0.016 for “therapist_we”; r = −0.446, p = 0.017, for “therapist_i”; r = −0.424, p = 0.024 for “patient_i”. LIWC features (%) were logarithmically transformed (a.u. = artificial unit). Shades indicate 95% confidence level interval for predictions from a linear regression.

**Figure 2:**
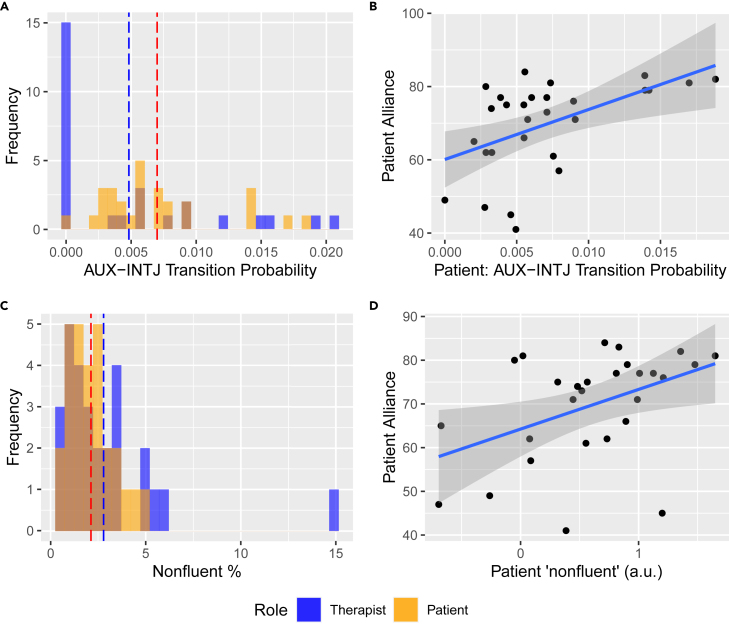
Patient’s non-fluency speech markers positively correlate with therapeutic alliance (A and C) Frequency distributions of AUX-INTJ (auxiliary verb-interjection, e.g. “is like”) transition probability, and “non-fluent” category (%) extracted from LIWC dictionary between speaker roles. Dotted vertical lines indicate the group means (mean = 0.005, s.e. = 2.33e-4 for “therapist_AUX-INTJ”; mean = 0.007, s.e. = 1.67e-4 for “patient_AUX-INTJ”; mean = 2.79, s.e. = 0.100 for “therapist_non-fluent”; mean = 2.12, s.e. = 0.041 for “patient_non-fluent”). (B and D) Patients’ AUX-INTJ probabilities, controlled for duration of treatment, and patients’ “non-fluent” feature show positive association with patient-rated alliance (ρ = 0.421, p = 0.029 for “patient_AUX-INTJ”; r = −0.437, p = 0.020, for “patient_non-fluent). “non-fluent” features (%) were logarithmically transformed (a.u. = artificial unit). Shades indicate 95% confidence level interval for predictions from a linear regression.

We identified three statistically significant pronoun features that regressed to patient-rated alliance, “therapist_we”, “therapist_i”, “patient_i” (p < 0.05, corrected). F-metrics for all significant features are listed in Table S1. Therapists spoke “we” words (e.g. we, our, us, let’s) 0.48% more than patients (t = 3.2, p = 0.003) and “i” words (e.g. I, me, my, myself) 7.0% less than patients (t = −19, p < 0.001) in their speech (Figures 1A and 1C). The frequency of “therapist_we” was negatively correlated with patient alliance (r = −0.45, p < 0.02) (Figure 1B), driven by personality disorder subgroup (n = 15, r = −0.58, p = 0.02) (Figure S2). “therapist_i” and “patient_i” were both negatively correlated with alliance (r = −0.45, p < 0.02, r = −0.42, p = 0.02) (Figure 2D), driven by non-personality disorder subgroup (n = 13, r = −0.64, p = 0.02, r = −0.61, p = 0.03) (Figure S2). “Patient_i” correlation effect size with bond subscore (r = −0.52) was significantly larger than with goal subscore (r = −0.29; t = 2.3, p = 0.03) (Figure S2).

Next, the semantic contexts of first-person pronoun usage were further disambiguated at the level of bigram (two-word) features containing “i” or “we.” Patients’ “when i,” and therapists’ “i do”, and “i think” were the most frequently detected items that were regressed against patient-rated alliance (F = 9.4, p = 0.02; F = 6.6, p = 0.02; F = 6.2; p = 0.02, respectively, corrected). The latter two were also significantly regressed against therapist-rated alliance (Table S1). Table 1 demonstrates examples of sentences in which individuals used first-person pronouns.

**Table 1:** Examples of sentences that contained significant natural language processing features for patient-rated alliance

Speaker: Feature	Example Sentences
Therapist: i_do	***i do****understand the frustration you’re you’re having* (p = 41, T = 46)
	*it’s almost like****i do****want to know if it’s like something related to medications although you have been on it for some time now* (p = 45, T = 49)
Therapist: i_think	***i think****you have to remind yourself okay what is within my self control and what is not and****i think****that a lot of the things you’re worrying about* (p = 47, T = 51)
	*maybe but****i think****it might be something more significant than that* (p = 57, T = 40)
Therapist: we	*thanks for being honest with me about that that’s really important for****our****work now****let’s****talk about that* (p = 45, T = 49)
	*right now what****we’ve****been doing is really trying to take you what are the thoughts that you’ve been having* (p = 75, T = 73)
Patient: when_i	***when i****brought up my frustrations about here where he’s been like please give it give it a go give it a go and****when i****talk to you* (p = 41, T = 46)
Patient: AUX-INTJ (auxiliary verb-interjection transition)	*and they****were like****no they can accommodate you and i****was like****they better cause i’m i’m gonna lose it i don’t wanna think that way* (p = 81, T = 82)
Patient: AUX-INTJ and INTJ-PRON (interjection-pronoun transition)	*it’s incredibly dangerous job with very inhumane conditions and the pay is not nearly what it should****be um****and i never kind of****like i****understood it was dangerous* (p = 84, T = 77)
Patient: AUX-INTJ (talk-turn interaction)	*Patient*: *i figure i might as well deal with as much of it as i****can*** *Therapist*: ***yeah*** (p = 71, T = 77)
Patient: non-fluent	*Patient*: ***uhh****snowstorm and that sort of thing*, *yes*. *Therapist*: *yeah*, *yeah* *Patient*: ***umm***, *otherwise****ehhh****things have been going*, *moderately well i suppose* (p = 81, T = 61)

Each sentence is annotated with the P = alliance score rated by patient, T = alliance score rated by therapist.

### Relationship between non-fluency and alliance rating

In terms of non-fluency, we found four significant features related to interjection part-of-speech transition, patients’ auxiliary verb—interjection (AUX-INTJ, e.g. am like), adverb—interjection (ADV-INTJ, e.g. just like), interjection—pronoun (INTJ-PRON, e.g. uh i) and therapists’ interjection—subordinating conjunction (INTJ-SCONJ, e.g. um that) (p < 0.05, corrected) (Table S1). The AUX-INTJ feature took place more frequently in patients than in therapists (Wilcoxon r = 0.29, p = 0.03) (Figure 2A). Patients’ AUX-INTJ transition was positively correlated with alliance after controlling for duration of treatment (ρ = 0.42, p = 0.03) (Figure 2B). The AUX-INTJ feature, which marked non-fluency within speech, also identified the end of patients’ utterance transitioning to therapists’ acknowledgment words (e.g. “could. Oh”, “are. Yeah”) (Table 1). The ‘“non-fluent” speech metric (e.g. hmm, um) did not differentiate between speaker roles (t = −0.58, p = 0.56) (Figure 2C), but only the patient feature was positively correlated with alliance (r = 0.44, p < 0.02) (Figure 2D). AUX-INTJ and the “non-fluent” speech metric neither had a higher correlation with any specific alliance subscores nor regressed to therapist-rated alliance (Table S1).

### Relationship between trust game behavior and language features

**Figure 3:**
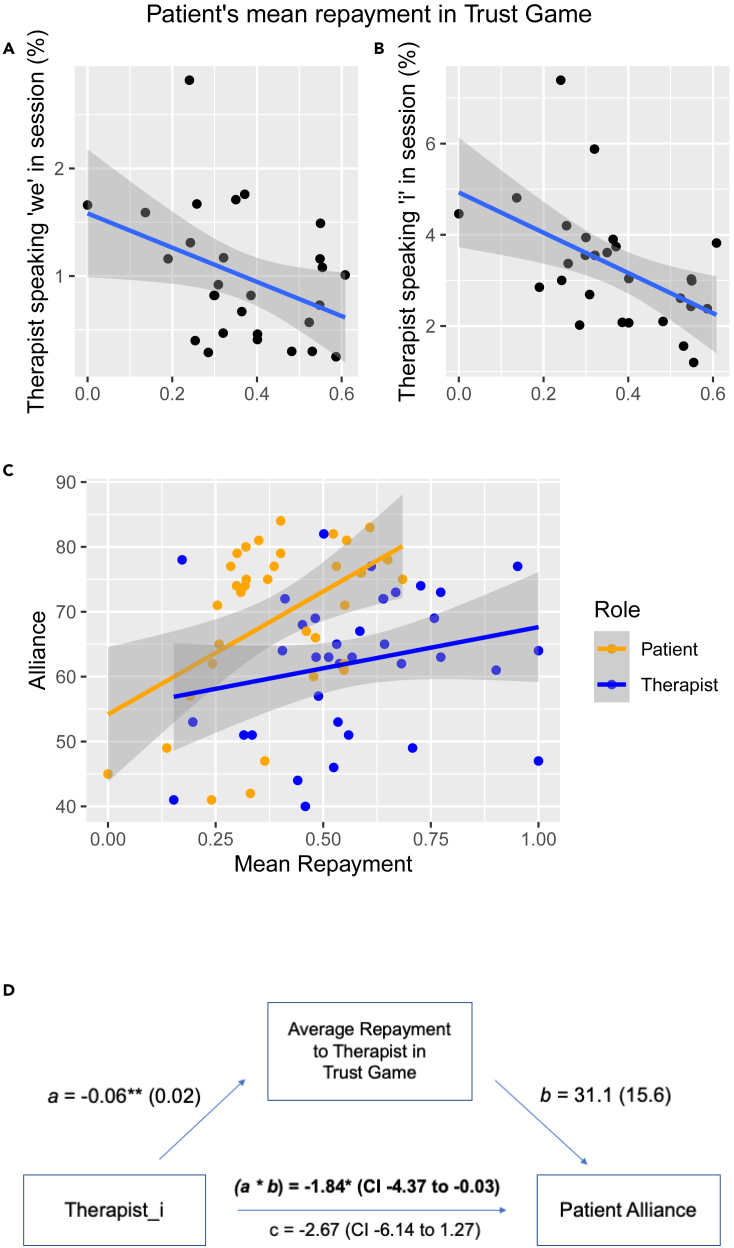
Patients’ mean repayment fractions to therapists in the trust game negatively associate with the therapists’ first-person pronoun frequencies extracted from the sessions (A and B) Patients (n = 28) had larger mean repayment fraction ratios when their investors in the game, i.e. therapists, spoke less first-person pronouns (“we” and “i”) (r = −0.376, p = 0.049 for “therapist_we”; r = −0.532, p = 0.004 for “therapist_i”). (C) Patients had mean repayment fraction ratios proportional to their therapeutic alliance scores with the investors (r = 0.482, p = 0.003), whereas therapists did not with their investors (r = 0.237, p = 0.158). (D) Mean repayment fraction across 10 rounds statistically mediated the association between “therapist_i” and patient-reported alliance, suggesting a potential mechanism in which therapist’s language recruits a trusting behavior to impact alliance. Arrows indicate the direction of linear regressions, annotated with coefficient estimates (standard error) and 95% confidence intervals ∗p < 0.05, ∗∗p < 0.01.

Finally, patients’ average repayment fractions toward therapists in the trust game were negatively correlated with their therapists’ frequency of speaking “we” and “i” in the sessions (r = −0.38, p = 0.05; r = −0.53, p = 0.004) (Figures 3A and 3B). Patients’ average repayment fractions toward therapists were positively correlated with self-reported alliance ratings (r = 0.48, p = 0.003), whereas therapists’ average repayment fractions toward patients were not (Figure 3C). Patients’ repayment fractions were not significantly correlated with their AUX-INTJ transition probabilities (ρ = 0.37, p = 0.06). We also explored whether negative correlations of therapists’ first-person pronoun usage with alliance were mediated by repayment behavior. The mediation analysis indicated a significant effect of the indirect path for “therapist_i” (a ∗ b = −1.84, p = 0.04, 95% CI = −4.37 to −0.03), but not for “therapist_we” (a ∗ b = −3.05, p = 0.05, 95% CI = −7.50 to 0.01), indicating that the patients’ perceived trustworthiness of the therapist—as measured by their repayment toward the therapist in the trust game—mediated the effect of therapists’ first-person singular pronoun use on patient-reported alliance (Figure 3D).

## Discussion

**Figure 4:**
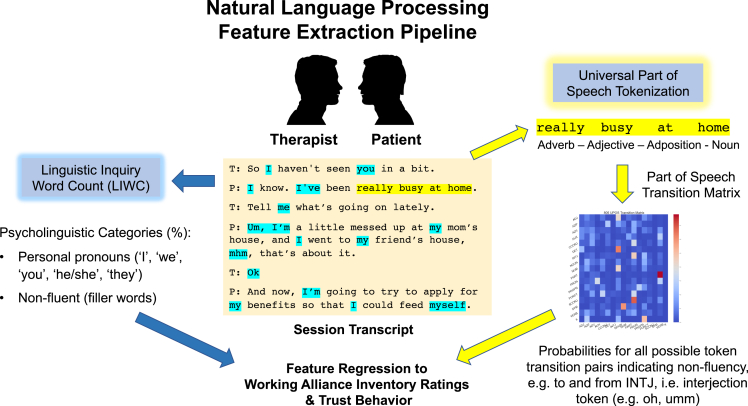
Diagram of natural language processing (NLP) feature extraction pipeline Audio files of psychotherapy sessions were transcribed and de-identified for personally identifiable information. Linguistic features were generated individually from a given session transcript, i.e. personal pronoun usage metric from the linguistic inquiry word count (LIWC) dictionary and part-of-speech (POS) transition probabilities following word tokenization. The NLP feature frequencies were regressed to alliance ratings, which were completed post-session by therapists and patients.

Scalable, yet interpretable markers of patient-therapist alliance in naturalistic psychotherapy sessions can provide timely and clinically actionable feedback in mental health treatment. Here, we analyzed personal pronoun usage and non-fluency markers using feature extraction methods commonly used in NLP, combined with self-reported surveys of alliance and a game theoretic approach toward alliance (i.e. trust game) (Figure 4). Our study provides the first computational evidence that both first-person pronoun and non-fluency are potential language markers that are predictive of therapeutic alliance and interpersonal trust during psychotherapy treatment.

Our primary finding was that more frequent first-person pronoun usage in both therapists and patients (“we,” “i do,” “i think”, “when i”) characterized sessions with lower alliance ratings by patients cross-sectionally, consistent with the first hypothesis. Sentences containing these features were largely statements disclosing their thoughts and emotions. In a treatment context where patients predominantly expressed themselves (“i”) (Figure 1C), therapists’ expression of “i,” especially with cognitively geared verbs (e.g. “i do”, “i think”), may have signaled their inadequate responsiveness to patients’ emotional needs, which has been associated with negative treatment outcomes.^36^ In terms of “we,” one might assume that the usage of such pronoun that signals inclusiveness might correlate with higher, rather than lower alliance. However, when used by therapists, a higher frequency of “we” could have indicated their therapeutic techniques to bring the strained relationships the patients were dealing with into the “we” mode of togetherness.^37^^,^^38^ These speech features not only correlated with patients’ perception of alliance, i.e. self-report, but also with objectively measured behavioral proxies of trust (Figures 3A–3C). Expanding the previous findings from the trust game literature in which patients repaid higher amounts to clinicians with good communication skills,^35^ we also identified a role of interpersonal trust behavior in mediating the negative relationship between the therapist’s use of “i” and patient-rated alliance (Figure 3D). This result further revealed that the therapist’s self-expression might negatively influence alliance through direct cognitive changes related to interpersonal processing in the patient.

Regarding the second hypothesis, we found that higher non-fluency in patients (e.g. “is like,” “umm”), but not in therapists, characterized sessions with higher alliance ratings by patients (Figures 2B and 2D). In natural language, honest and emotionally regulated speech often contains non-fluency.^26^^,^^27^ This offers a plausible explanation for our finding that in our sample, patients who reported stronger alliance were more honest and more willing to effectively communicate their emotions to their therapists. The probability of the auxiliary verb token transitioning to the filler word token also identified the end of patients’ sentences being acknowledged by therapists (e.g. “could. Oh”, “are. Yeah”). This finding was consistent with previous work demonstrating that interpersonal attunement measured from behavioral coordination^6^^,^^7^^,^^8^^,^^9^^,^^25^ predicted alliance during treatment.

### Limitations of the study

Our results should be interpreted with the following caveats. First, this is an observational study, which does not provide causal or mechanistic insight into the relationship between linguistic patterns and therapeutic alliance. Future studies that incorporate interventional and/or longitudinal designs (e.g. clinical trial) might be able to address causality by examining the effects of language stimuli with or without these language features on therapeutic outcomes. Second, we did not analyze the immediate context in which the features were discovered. It is possible that the use of personal pronouns and non-fluency could have different meanings depending on the clinical context they were said. Analysis of non-verbal features (e.g. voice acoustics) and/or domain expert annotation could identify such context. Third, the fidelity of paralinguistic markers (e.g. “umm”) may be limited due to the imperfect nature of human transcription, despite being the current gold standard. State-of-the-art automatic speech recognition technology that can transcribe paralinguistic markers/disfluencies with good accuracy against human transcription can significantly address this methodological limitation, especially when text analysis is done at scale. Finally, the sample size is small, thus limiting statistical power to detect significant relationships between alliance and other language features outside of our main hypotheses. Nevertheless, this study provides important initial insight that could lay the foundation for larger-scale studies to replicate existing findings and identify additional interpretable and predictive language features of alliance.

In summary, our NLP approach revealed first-person pronoun and non-fluency features as clinically relevant markers of alliance from psychotherapeutic dialogues. As psychotherapy begins to integrate more technology (e.g., teletherapy and text-based therapy), computational analysis of patient-clinician interactions can be a fruitful avenue for elucidating key elements that make treatment effective at scale.

## STAR★Methods

### Key resources table

**Table:** 

REAGENT or RESOURCE	SOURCE	IDENTIFIER
**Deposited data**		

Language features and trust game dataset	This paper	https://github.com/iamj1han/iScience_therapy_alliance

**Software and algorithms**		

LIWC-2015	Pennebaker Conglomerates, Inc	https://www.liwc.app
Transcript preprocessing, feature generation, and statistical analysis codes	This paper	https://github.com/iamj1han/iScience_therapy_alliance
R	R Foundation for Statistical Computing	https://www.r-project.org
Python	Python Software Foundation	https://www.python.org

### Resource availability

#### Lead contact

Jihan Ryu (jihan.i.ryu@gmail.com) and Xiaosi Gu (xiaosi.gu@mssm.edu).

#### Materials availability

This study did not generate new unique reagents.

### Experimental model and study participant details

In this cross-sectional study, we recorded twenty-eight single sessions from ongoing psychotherapy treatment (patient n=28, therapist n=18) in two academic hospital-based outpatient clinics, one general adult psychiatric clinic and one personality disorder-specialized clinic in New York City from February 2020 to November 2021. The median age in our patient sample was 39 years old, with a range of 19 to 64 years (SD 15). 20 (71%) of the subjects were female, and 6 (21%) were receiving treatment in the personality disorder clinic. The most commonly represented psychiatric diagnosis was personality disorder 15 (54%, 12 borderline personality, 3 narcissistic personality), followed by mood disorder 7 (25%, 5 unipolar and 1 bipolar depressive disorders, 1 unspecified mood disorder), and anxiety and trauma-related disorders 6 (21%, 2 generalized anxiety disorder, 4 post-traumatic stress disorder). All patients had previous therapy experience with a median number of lifetime therapists of 5, ranging from 2 to 15 (SD 3.8). 12 (71%) of the therapists were female. 6 (25%) were faculty psychologists, and 11 (75%) were trainee psychologists and psychiatrists. All therapists provided non-manualized psychotherapy with supportive and relationally oriented techniques, 24 (86%), or cognitive-behaviorally oriented techniques, 4 (14%), to improve their patients’ interpersonal functioning. Eight therapists provided more than one session with different patients in the sample. At the time of recording, patients were at a median of 14.5^th^ session, with a range of 2 to 160 sessions (SD 32). The exclusionary criteria included use of a non-English language during therapy sessions and presence of neurological or other conditions that affect perception and expression of language. Written informed consent and our protocol were approved by the Institutional Review Board at Icahn School of Medicine at Mount Sinai.

### Method details

#### Recording and clinical assessment procedures

Prior to sessions, we used an online survey to assess patients’ alliance with a previous therapist on a 10-point Likert scale (1-negative, 10-positive) and attachment-related avoidance and anxiety traits using Revised Adult Attachment Scale,^39^ factors that can influence therapeutic alliance. Therapy sessions were recorded using two wireless microphones, which were clipped on their clothes to facilitate separation and transcription of individual speech, and the backup physical recorder. Subjects sat 6 feet apart for in-person sessions, or over teletherapy applications for remote sessions, in the room. After recorded sessions, patients and therapists privately completed the Working Alliance Inventory - Short Form (WAI-SR) (Reprinted with permission of Society for Psychotherapy Research).^40^ Patients and therapists each rated the 12 items on a 7-point Likert scale ranging from "Never" to "Always,” providing results in three domains: (a) agreement about goals of the treatment (goal); (b) agreement about the tasks to achieve treatment goals (task); and (c) the bond quality between therapist and patient (bond).

#### Trust game

We administered a 10-round version of the trust game in which all subjects played the “trustee” role with the computer-simulated investor to match the overall strategy of the subject’s partner.^41^ The subject was instructed to play as if the investor were the real-life therapy partner. In each round, the investor offered to the subject a portion of 20 monetary units. The initial offer was random, and subsequent offers were chosen from an independent dataset of players’ choices from the same round using a k-nearest neighbors sampling algorithm.^42^ The offer was tripled and sent to the trustee (subject), who decided how much to repay out of that amount. The repayment fraction was averaged across responses in all ten rounds (RF_t_ = R_t_ / I_t_, where R = trustee’s repayment amount, I = amount received at round t) and analyzed as a proxy of trustworthiness of the investor (therapy partner).^43^^,^^44^ No specific goals for the game were provided, but the subjects had incentives to maximize the cumulative amount they had kept for themselves after ten rounds, since it increased their bonus reimbursement in proportion.

#### Natural language processing algorithms for transcripts

Audio files were transcribed independently by two researchers (JR and CM) into lines of text, followed by separation of speaker roles (i.e., patient and therapist) and removal of personally identifying information and punctuation markers (See key resources table for text preprocessing code). For natural language processing feature extraction methods, we used Linguistic Inquiry Word Count (LIWC, 2015 edition)^45^ and Part of Speech (POS) tokenization.^46^ LIWC is a computerized, word count-based text analysis software tool that maps each word into psycholinguistic categories. The entire set of LIWC features were extracted, but only the first, second, and third personal pronoun, as well as ‘non-fluent’ category word usage frequencies scaled by the total word counts spoken by each speaker in the session were considered for statistical analysis. Another common language technique that has been used to characterize speaking style of a patient or therapist in the previous literature is n-grams (a single word or short, multi-word phrases in length of n).^11^^,^^13^^,^^47^ N-gram based models characterize language use as the probability of speaking any word at the present instance, given the preceding word or words. We first encoded a sequence of all words spoken by each speaker in the session with Part-Of-Speech tagging, a tokenization method that assigns each word a syntactic label (e.g. ADJ: adjective, AUX: auxiliary, INTJ: interjection). To extract an independent metric that characterizes non-fluency as a multi-phrase phenomenon, which complements the LIWC dictionary-based “non-fluent” frequency, we calculated all syntactic transition probabilities between any two contiguous tokens (bi-grams), and considered those that start or end with INTJ (interjection; “uh,” “oh,” “hmm”) as non-fluency metrics, generating POS transition matrix. For example, in the case of X-INTJ-Y tokenized sequences, X-INTJ and INTJ-Y transition probabilities were calculated as separate features (See Figure 4 for the data processing pipeline). POS tagging was accomplished with Stanza package.^48^ Bi-grams and transition matrix were generated with Python code written for this project (See key resources table).

### Quantification and statistical analysis

The personal pronoun and bi-gram transition probability features were linearly regressed against alliance scores using F-test for statistical significance (false discovery rate corrected, α = 0·05). The LIWC feature frequency differences between speaker roles and pairwise correlations with alliance (self-reported ratings and trust game average repayment fraction) were summarized using a paired t-test and Pearson’s r after logarithmically transforming the values. For transition probabilities, which were highly skewed towards zero, we used Wilcox rank sum test and Spearman’s ρ, respectively.^49^ Alliance scores were compared using Kruskal-Wallis one-way ANOVA across categorical clinical variables. Partial correlation corrected by the duration of treatment (only for AUX-INTJ, due to its correlation with treatment duration, which was logarithmically transformed; see Figure S2), and Steiger’s Z test to examine differences in dependent correlations (feature ∼ subscores of alliance, i.e. goal, task, bond), were performed using ‘psych’ R package.^50^

For exploratory analysis testing if the average repayment fraction from trust game mediates the association between therapist’s first person pronoun features and alliance ratings, we used ‘mediation’ R package.^51^ We estimated confidence intervals in the effects of mediation using a quasi-Bayesian approximation approach (1,000 iterations, α = 0.05) and considered the mediation significant if the total indirect effect (a ∗ b) was statistically significant, while the previously significant direct effect (path c) became non-significant after controlling for the mediator. All statistical analyses were conducted with two-sided Type I error of 5%. Python 3.8.5, Stanza 1.2, and RStudio 2022.02.3 were used for analysis.

## Data Availability

• De-identified datasets have been deposited at a publicly available repository as of the date of publication. DOIs are listed in the key resources table. The full transcript data in this study cannot be deposited in a public repository because these are withheld by the corresponding author’s institution IRB to preserve patient and therapist privacy and confidentiality. • All original code has been deposited at a publicly available repository as of the date of publication. DOIs are listed in the key resources table. • Any additional information required to reanalyze the data reported in this paper is available from the lead contact upon request.

## References

[1] Norcross J.C., Lambert M.J. (2018). Psychotherapy relationships that work III. Psychotherapy.

[2] Horvath A.O. (2018). Research on the alliance: knowledge in search of a theory. Psychother. Res..

[3] Shimokawa K., Lambert M.J., Smart D.W. (2010). Enhancing treatment outcome of patients at risk of treatment failure: meta-analytic and mega-analytic review of a psychotherapy quality assurance system. J. Counsel. Psychol..

[4] Flemotomos N., Martinez V.R., Chen Z., Singla K., Ardulov V., Peri R., Caperton D.D., Gibson J., Tanana M.J., Georgiou P. (2022). Automated evaluation of psychotherapy skills using speech and language technologies. Behav. Res. Methods.

[5] Ryu J., Banthin D.C., Gu X. (2021). Modeling therapeutic alliance in the age of telepsychiatry. Trends Cognit. Sci..

[6] Ramseyer F., Tschacher W. (2014). Nonverbal synchrony of head- and body-movement in psychotherapy: different signals have different associations with outcome. Front. Psychol..

[7] Ellingsen D.M., Isenburg K., Jung C., Lee J., Gerber J., Mawla I., Sclocco R., Jensen K.B., Edwards R.R., Kelley J.M. (2020). Dynamic brain-to-brain concordance and behavioral mirroring as a mechanism of the patient-clinician interaction. Sci. Adv..

[8] Tschacher W., Meier D. (2020). Physiological synchrony in psychotherapy sessions. Psychother. Res..

[9] Zhang Y., Meng T., Hou Y., Pan Y., Hu Y. (2018). Interpersonal brain synchronization associated with working alliance during psychological counseling. Psychiatry Res. Neuroimaging..

[10] Sened H., Zilcha-Mano S., Shamay-Tsoory S. (2022). Inter-brain plasticity as a biological mechanism of change in psychotherapy: a review and integrative model. Front. Hum. Neurosci..

[11] Goldberg S.B., Flemotomos N., Martinez V.R., Tanana M.J., Kuo P.B., Pace B.T., Villatte J.L., Georgiou P.G., Van Epps J., Imel Z.E. (2020). Machine learning and natural language processing in psychotherapy research: alliance as example use case. J. Counsel. Psychol..

[12] Goldberg S.B., Tanana M., Imel Z.E., Atkins D.C., Hill C.E., Anderson T. (2021). Can a computer detect interpersonal skills? Using machine learning to scale up the Facilitative Interpersonal Skills task. Psychother. Res..

[13] Zech J.M., Steele R., Foley V.K., Hull T.D. (2022). Automatic rating of therapist facilitative interpersonal skills in text: a natural language processing application. Front. Digit. Health.

[14] Tsakalidis A., Atzil-Slonim D., Polakovski A., Shapira N., Tuval-Mashiach R., Liakata M. (2021). Proceedings of the Seventh Workshop on Computational Linguistics and Clinical Psychology: Improving Access.

[15] Cadario R., Longoni C., Morewedge C.K. (2021). Understanding, explaining, and utilizing medical artificial intelligence. Nat. Hum. Behav..

[16] Ackerman S.J., Hilsenroth M.J. (2003). A review of therapist charcteristics and techniques positively impacting the therapeutic alliance. Clin. Psychol. Rev..

[17] Nook E.C., Schleider J.L., Somerville L.H. (2017). A linguistic signature of psychological distancing in emotion regulation. J. Exp. Psychol. Gen..

[18] Nook E., Hull T.D., Nock M.K., Somerville L.A.-O. (2022). Linguistic measures of psychological distance track symptom levels and treatment outcomes in a large set of psychotherapy transcripts. Psychol. Cognit. Sci..

[19] Leis A., Ronzano F., Mayer M.A., Furlong L.I., Sanz F. (2019). Detecting signs of depression in tweets in Spanish: behavioral and linguistic analysis. J. Med. Internet Res..

[20] Todorov G., Mayilvahanan K., Cain C., Cunha C. (2020). Context- and subgroup-specific language changes in individuals who develop PTSD after trauma. Front. Psychol..

[21] Kelley S.W., Mhaonaigh C.N., Burke L., Whelan R., Gillan C.M. (2022). Machine learning of language use on Twitter reveals weak and non-specific predictions. NPJ Digit. Med..

[22] Van Staden C.W., Fulford K.W.M. (2004). Changes in semantic uses of first person pronouns as possible linguistic markers of recovery in psychotherapy. Aust. N. Z. J. Psychiatr..

[23] Martinez V., Flemotomos N., Ardulov V., Somandepalli K., Goldberg S., Imel Z., Atkins D., Narayanan S. (2019). Identifying therapist and client personae for therapeutic alliance estimation. Interspeech.

[24] Nienhuis J.B., Owen J., Valentine J.C., Winkeljohn Black S., Halford T.C., Parazak S.E., Budge S., Hilsenroth M. (2018). Therapeutic alliance, empathy, and genuineness in individual adult psychotherapy: a meta-analytic review. Psychother. Res..

[25] Koole S.L., Tschacher W. (2016). Synchrony in psychotherapy: a review and an integrative framework for the therapeutic alliance. Front. Psychol..

[26] Villar G., Castillo P. (2017). The presence of 'um' as a marker of truthfulness in the speech of TV personalities. Psychiatr. Psychol. Law.

[27] Roche J.M., Arnold H.S. (2018). The effects of emotion suppression during language planning and production. J. Speech Lang. Hear. Res..

[28] Oomen C.C., Postma A. (2001). Effects of divided attention on the production of filled pauses and repetitions. J. Speech Lang. Hear. Res..

[29] Lord S.P., Sheng E., Imel Z.E., Baer J., Atkins D.C. (2015). More than reflections: empathy in motivational interviewing includes language style synchrony between therapist and client. Behav. Ther..

[30] Xiao B., Imel Z.E., Atkins D.C., Georgiou P.G., Narayanan S.S. (2015). Analyzing speech rate entrainment and its relation to therapist empathy in drug addiction counseling. Proc. Interspeech.

[31] Schaper R., Nowotny C., Michalek S., Schmidt U., Brockmeyer T. (2023). Language style matching and treatment outcome in anorexia nervosa. Eur. Eat Disord. Rev..

[32] Wadden D., August T., Li Q., Althoff T. (2021).

[33] Camerer C. (2003).

[34] Kreps D. (1990).

[35] Kovacs R.J., Lagarde M., Cairns J. (2019). Measuring patient trust: comparing measures from a survey and an economic experiment. Health Econ..

[36] Anderson T., Bein E., Pinnell B., Strupp H. (1999). Linguistic analysis of affective speech in psychotherapy: a case grammar approach. Psychother. Res..

[37] Eubanks C.F., Lubitz J., Muran J.C., Safran J.D. (2019). Rupture resolution rating system (3RS): development and validation. Psychother. Res..

[38] Choi-Kain L.W., Simonsen S., Euler S. (2022). A mentalizing approach for narcissistic personality disorder: moving from "Me-Mode" to "We-Mode". Am. J. Psychother..

[39] Collins N.L., Read S.J. (1990). Adult attachment, working models, and relationship quality in dating couples. J. Pers. Soc. Psychol..

[40] Tracey T.J., Kokotovic A.M. (1989). Factor structure of the working alliance inventory. Psychol. Assess.: J. Consult. Clin. Psychol..

[41] King-Casas B., Tomlin D., Anen C., Camerer C.F., Quartz S.R., Montague P.R. (2005). Getting to know you: reputation and trust in a two-person economic exchange. Science.

[42] IBM (2022). k-nearest neighbors algorithm (KNN). https://www.ibm.com/docs/en/ias?topic=procedures-k-nearest-neighbors-knn.

[43] Alós-Ferrer C., Farolfi F. (2019). Trust games and beyond. Front. Neurosci..

[44] Tzieropoulos H. (2013). The Trust Game in neuroscience: a short review. Soc. Neurosci..

[45] Pennebaker J.W., Ryan L.B., Jordan K., Blackburn K. (2015).

[46] Jurafsky D.M., James H. (2000).

[47] Xiao B., Imel Z.E., Georgiou P.G., Atkins D.C., Narayanan S.S. (2015). "Rate my therapist": automated detection of empathy in drug and alcohol counseling via speech and Language Processing. PLoS One.

[48] Qi P., Zhang Y., Zhang Y., Bolton J., Manning Christopher D. (2020). Proceedings of the 58th Annual Meeting of the Association for Computational Linguistics: System Demonstrations.

[49] de Winter J.C.F., Gosling S.D., Potter J. (2016). Comparing the Pearson and Spearman correlation coefficients across distributions and sample sizes: a tutorial using simulations and empirical data. Psychol. Methods.

[50] Revelle W. (2022). Psych: procedures for psychological, psychometric, and personality research. https://personality-project.org/r/psych/.

[51] Tingley D., Yamamoto T., Hirose K., Keele L., Imai K. (2014). Mediation: R package for causal mediation analysis. J. Stat. Softw..

