# Supplementary material for: A natural language processing approach reveals first-person pronoun usage and non-fluency as markers of therapeutic alliance in psychotherapy
Source: iScience. 2023 May 12;26(6):106860. doi: 10.1016/j.isci.2023.106860 (PMC10225921; doi:10.1016/j.isci.2023.106860)
Supplement: Document S1. Figures S1 and S2 and Table S1 [file mmc1.pdf]

iScience, Volume *26*

Supplemental information

A natural language processing approach reveals first-person pronoun usage and non-fluency as markers of therapeutic alliance in psychotherapy

Jihan Ryu, Stephen Heisig, Caroline McLaughlin, Michael Katz, Helen S. Mayberg, and Xiaosi Gu

Supplemental Information

Figure S1. Summary of relationships between therapeutic alliance (n=28) and clinical variables, related to Alliance Ratings in Results.

(A) Patient-rated alliance was positively correlated with therapist-rated alliance (r=0.56, p<0.002), and therapist-rated alliance was positively correlated with patient's avoidant attachment scores (r=0.40, p=0.04). Patient alliance was not correlated with duration of treatment (i.e. number of visits with the current therapist), attachment scores, or age (p>0.05). (B-F) Violin diagrams indicating distribution of alliance by sex, diagnoses, therapist experience, modalities of treatment, and use of telehealth. Center lines indicate median alliance in the sub-groups. There were no significant differences in alliance across the sub-groups (p>0.05). Significance in the alliance correlations was tested with Pearson's for continuous variables, and Wilcox rank sum test and Kruskal-Wallis one way ANOVA for categorical variables with two-sided Type I error of 5%.

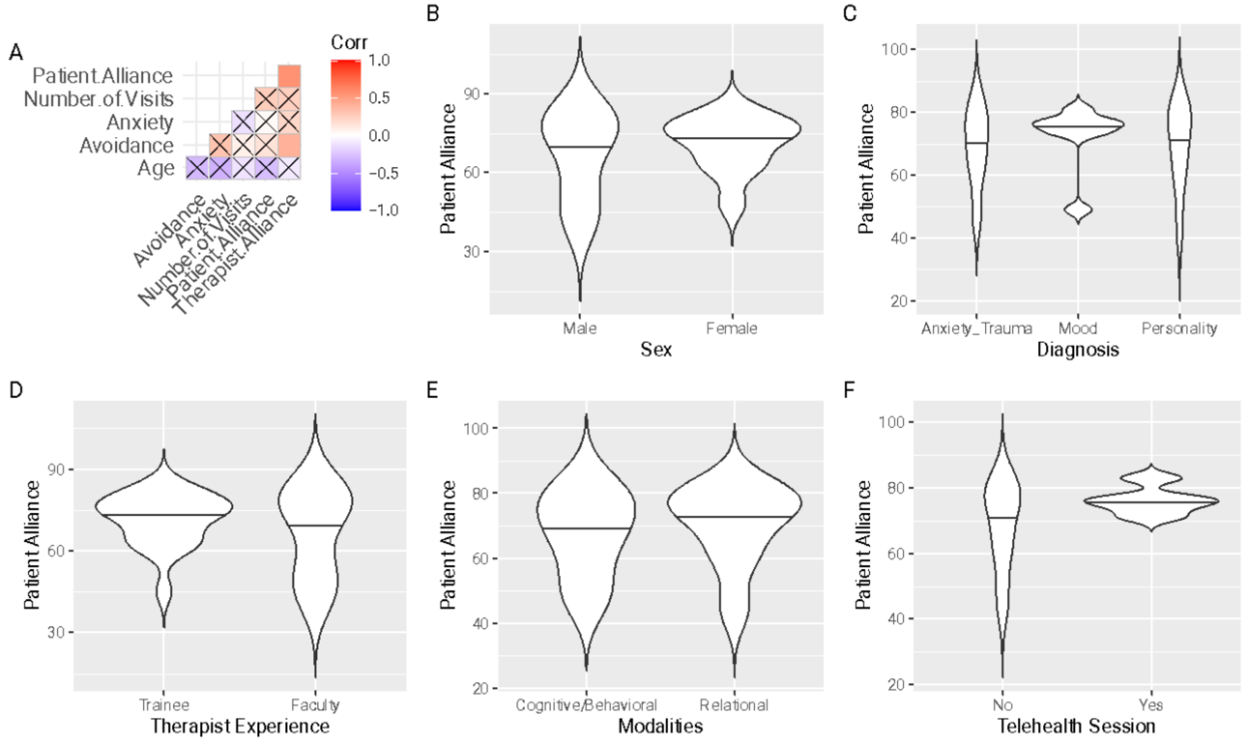

Figure S2. Correlation of linguistic features with alliance across diagnostic subgroups of patients, subscores of alliance, and duration of treatment, related to Figure 1 and 2.

(A) The negative correlation between frequency of “therapist_we” and patient alliance was driven by personality disorder (n=15, r=-0.58, p=0.02) versus non-personality disorder subgroup (n=13, r=-0.31, p=0.30). (B) “Patient_i” and “therapist_i” were both negatively correlated with alliance, driven by non-personality disorder (n=13, r=-0.64, p=0.02, r=-0.61, p=0.03) versus personality disorder subgroup (n=15, r=-0.23, p=0.40, r=-0.33, p=0.22). (C) The negative correlation of ‘patient_i” with bond subscore (r=-0.52) was significantly larger than with goal subscore (r=-0.29; t=2.27, p=0.03). Correlations with sub-scores of alliance (Goal, Task, Bond) were compared for pairwise significant differences using Steiger's Z test for each feature. (D) Patients’ AUX-INTJ transition probability was positively correlated with duration of treatment (ρ=0.41, p=0.03). Significance of correlation was tested using Spearman’s ρ. Duration of treatment (i.e. number of visits) was logarithmically transformed.

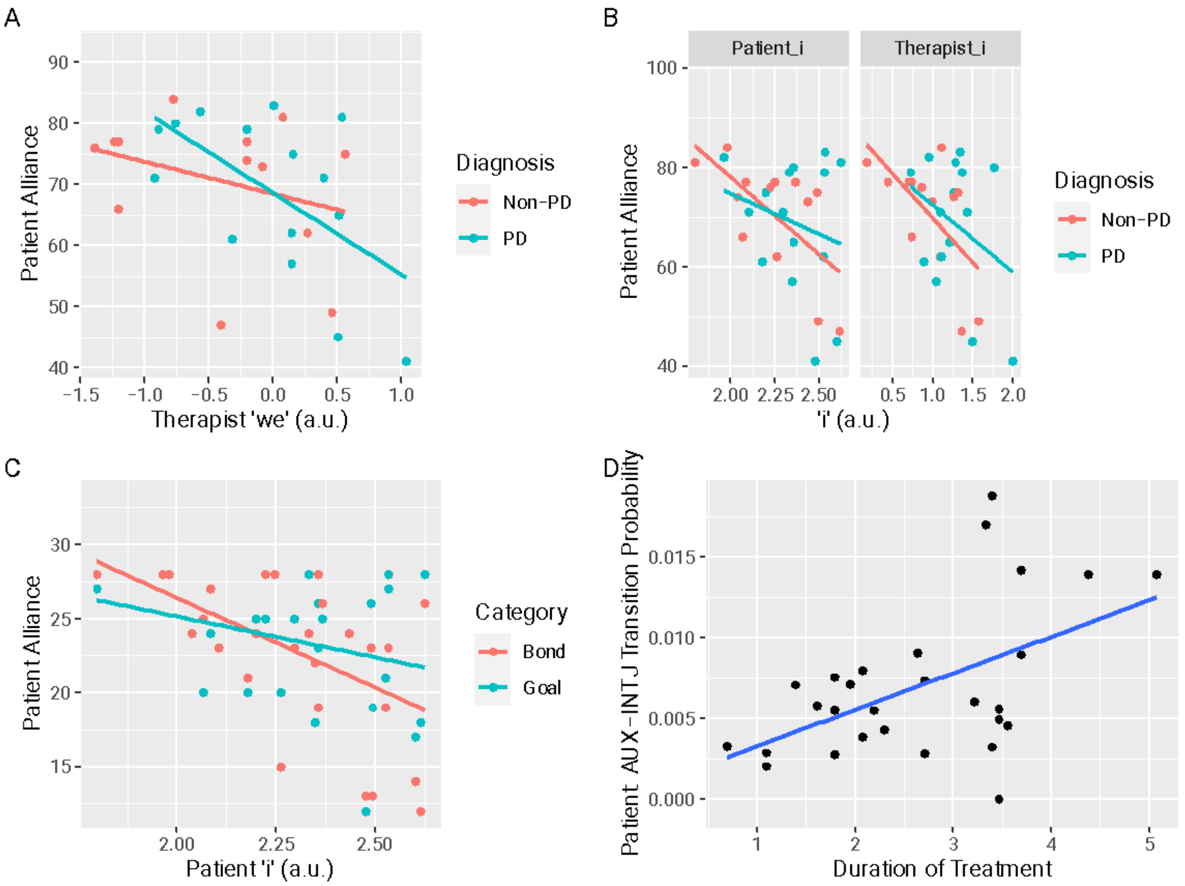

Table S1. The list of significant psycholinguistic features and their F-test metrics of linear regression on therapeutic alliance self-ratings, related to Relationship between pronouns/non-fluency and alliance rating in Results.

Part of Speech categories - AUX: Auxiliary, CCONJ: Coordinating conjunction, ADP: Adposition, ADV: Adverb, INTJ: Interjection, PRON: Pronoun, SCONJ: Subordinating conjunction. ‘we’ denotes all singular plural pronouns based on LIWC metric (“we”, ”our”, ”let’s”, etc), whereas ‘pronoun_we’ denotes only “we.” *p* values were with false-discovery rate corrected.

	Feature	*Regression: Patient Alliance*			*Regression: Therapist Alliance*	
		F-Test Score	*FT-p value*	*Corrected p-value*	*F-Test Score*	*FT-p value*
1	Therapist_we	10.02	0.0039	0.0225	2.403	0.1332
2	Patient_when_i	9.425	0.0050	0.0225	3.219	0.0844
3	Patient_AUX,INTJ	9.367	0.0051	0.0225	0.662	0.4231
4	Therapist_pronoun_we	9.110	0.0070	0.0225	2.395	0.1338
5	Therapist_i	7.616	0.0160	0.0261	0.430	0.5177
6	Therapist_i_do	6.643	0.0160	0.0277	4.332	0.0474
7	Patient_ADV,INTJ	6.487	0.0171	0.0277	0.013	0.9103
8	Patient_INTJ,PRON	6.438	0.0175	0.0277	1.349	0.2561
9	Therapist_i_think	6.215	0.0194	0.0277	7.630	0.0104
10	Patient_i	5.654	0.0251	0.0334	0.866	0.3608
11	Therapist_INTJ,SCONJ	4.834	0.0370	0.0392	4.290	0.0484
12	Patient_nonfluent	4.819	0.0372	0.0392	0.476	0.4964
